# Evaluation of Hyperlipasemia and Clinical Signs in 106 Dogs After Hospitalization for Acute Pancreatitis: Results From a Combined Retrospective and Prospective Follow‐Up Study

**DOI:** 10.1111/jvim.70188

**Published:** 2025-08-01

**Authors:** Peter H. Kook

**Affiliations:** ^1^ Clinic for Small Animal Internal Medicine, Vetsuisse Faculty University of Zurich Zurich Switzerland

**Keywords:** DGGR, dogs, follow‐up, lipase activity, pancreatitis, PLI, progression

## Abstract

**Background:**

No data after hospitalization for acute pancreatitis (AP) in dogs comparing clinical signs to lipase results exists.

**Objectives:**

Evaluate disease severity, lipase activity, and pancreatic lipase immunoreactivity (PLI) after hospitalization for suspected AP.

**Animals:**

One hundred and six client‐owned dogs with a minimum of one re‐check 2 weeks after hospitalization for AP.

**Methods:**

Combined retrospective and prospective study. Clinical signs graded using a clinical disease activity score (CDAS = CIBDAI complemented by abdominal pain) were compared to DGGR‐lipase activity (LIPC Roche) and PLI (SpecPL) at 2 weeks (*t*_2_, *n* = 106) after discharge. Additional re‐checks were available 6 weeks (*t*_3_, *n* = 56), 12 weeks (*t*_4_, *n* = 24), and 24 weeks (*t*_5_, *n* = 13) after discharge.

**Results:**

Lipase activity and PLI correlated strongly at all time points (*r*_s_ 0.863–0.937, *p* < 0.0001). Discordant results in regard to published reference intervals (RI) were rare (2.8% at *t*_2_, 1.7% at *t*_3_, 4.2% at *t*_4_, 0% at *t*_5_) and seemed clinically irrelevant. Dogs with still elevated lipase activity and PLI at *t*_2_ (24/106.22.6%) and *t*_3_ (21/56.37.5%) were significantly older compared to dogs with lipase within RI. Weak and moderate correlation between CDAS and lipase activity/PLI was found only at *t*_2_ (*r*_s_ 0.391, *p* = 0.0009; *r*_s_ 0.279, *p* = 0.004) and *t*_5_ (*r*_s_ 0.603, *p* = 0.032; *r*_s_ 0.57 *p* = 0.045). Most dogs (79.2%) with still elevated lipase at *t*_2_ had no or minimal clinical signs (CDAS 0–3). The same applied to all later re‐checks.

**Conclusion and Clinical Importance:**

Both lipase assays did not differ when compared to clinical status. Most dogs with hyperlipasemia after hospitalization for AP have no or minimal clinical signs.

**Abbreviations**
AP: acute pancreatitis
CDAS: clinical disease activity score
DGGR: 1,2‐o‐dilauryl‐rac‐glycero‐3‐glutaric acid‐(6 0‐methylresorufin) ester

## 1 Introduction

Acute pancreatitis (AP) is common in dogs. Similar to humans, the diagnosis is based on a combination of clinical, laboratory, and imaging findings depending on the acuity of illness [1, 2, 3]. Determination of serum lipase, measured either as a concentration (pancreatic lipase immunoreactivity [PLI]) [4] or activity (1,2‐o‐dilauryl‐rac‐glycero‐3‐glutaric acid‐(60‐methylresorufin) ester [DGGR]‐based lipase assays) [5, 6] is considered the laboratory test of choice. Both tests correlate strongly with each other (correlation coefficients in the studies vary between 0.89 and 0.96) [7, 8, 9, 10, 11, 12]. Sensitivities of the PLI test for a pancreatitis diagnosis vary depending on disease severity [13], while specificity is generally regarded as excellent compared to histopathology as the gold standard [1, 14]. PLI advocators have emphasized the allegedly poorer specificity of DGGR‐based lipase activity compared to the PLI assay [15], but in a clinical setting both assays (PLI using SpecPL, lipase activity using LIPC Roche) gave virtually the same results when lipase was measured daily in dogs hospitalized for AP [6]. So far, lipase has not been compared to the clinical status of dogs after recovery from AP. It remains unknown which test correlates better with the clinical condition of dogs after recovery from an AP episode.

Feeding a low‐fat diet during recovery from AP constitutes a standard supportive care [16]. It has been common practice in our hospital to re‐assess dogs after hospitalization for AP to see whether a low‐fat diet is still necessary. Since we repeatedly saw dogs with elevated lipase results with and without gastrointestinal clinical signs during these re‐checks, the author decided to investigate this in a more standardized way over a longer period of time.

The primary objective of this study was to investigate how frequently dogs have laboratory evidence of pancreatitis illustrated by elevated lipase activity and PLI 2 weeks after hospitalization for suspected AP. We also wanted to investigate which lipase test correlates better with the clinical condition of dogs after hospitalization for suspected AP. A secondary objective was to try to follow up on these dogs even further at standardized intervals. It was hypothesized that there would be no difference in the further course of the two lipase test values after discharge from the hospital and that there would be no difference compared to clinical signs.

## 2 Materials and Methods

### 2.1 Case Selection and Data Collection

Dogs that had been hospitalized for suspected AP were eligible for inclusion. Clinical diagnosis of AP was based on at least two of the following acute (≤ 7d duration) clinical signs (lethargy, abdominal pain, anorexia, vomiting, diarrhea), a lipase activity > 350 U/L (RI, 24–108 U/L) until 2022, and then > 450 U/L after renewal of the lipase activity RI to 17–156 U/L and/or a PLI > 400 μg/L (RI, 0–200), and absence of extra‐pancreatic diseases that could also explain clinical signs and hyperlipasemia. Lipase activity and PLI were always measured in serum.

All dogs had a standardized medical history, physical examination, CBC, biochemistry profile, and abdominal radiographs or abdominal ultrasonography (depending on individual case specifics) as minimum diagnostics performed. Exclusion criteria included dogs with major concurrent disease. Major concurrent disease included gastrointestinal obstruction, abdominal masses, cardiopulmonary disease, liver failure, metabolic disease, autoimmune disease, severe anemia, systemic infection, neoplastic disease, acute kidney injury, or chronic kidney disease IRIS stage > 2. Dogs with known comorbidities that could complicate further clinical assessment after discharge (e.g., any known chronic gastrointestinal disease requiring medical therapy, chronic hepatitis, hyperadrenocorticism) were also excluded. The decision to exclude dogs was based on the assessment of an experienced board‐certified internal medicine specialist.

Data for this study were obtained from retrospectively evaluated routine control visits (*n* = 65 dogs; 2015–2023) as well as from prospectively examined dogs (*n* = 41 dogs, 2020–2022). Cases were included if a complete data set was available from both the time of hospital admission (*t*_1_) and at least one re‐check visit 2 weeks after discharge (*t*_2_). A complete data set meant that all clinical information (medical history and physical examination) needed for the clinical disease severity score as well as results of both lipase activity and PLI had to be available. For the clinical assessment, a clinical disease activity score (CDAS) was used. The CDAS was based on the canine inflammatory bowel disease activity index (CIBDAI), which is an objective measure for assessing the severity of inflammatory bowel disease in dogs [17]. Because AP shares many clinical signs with IBD, we considered the CIBDAI a useful study tool to assess dogs with AP. Thus, the CIBDAI was complemented with additional information on suspected abdominal pain. Similarly modified CIBDAIs have already been used in other studies on pancreatitis in dogs [18, 19, 20, 21]. Presence of abdominal pain during first presentation (*t*_1_) was assessed based on history obtained from owners and clinical examination results and was graded as follows: 0 no abdominal pain detectable, 1 mild, 2 moderate, 3 severe pain. For follow‐up visits, presence of abdominal pain was categorized as 0 (no suspicion of abdominal pain by owners and based on clinical examination results) and 1 (dog owner thinks dog still has some abdominal pain or suspicion of abdominal pain during examination). For all re‐check visits, the remaining clinical signs of the CDAS were scored based on the history of 3 days before each visit. For the prospective part of the study, dog owners received a study plan at discharge that included planned follow‐up visits 2 weeks (*t*_2_), 6 weeks (*t*_3_), 12 weeks (*t*_4_), and 24 weeks (*t*_5_) after discharge. Each re‐check examination consisted of a standardized medical history, clinical examination, and blood sampling for measurement of lipase activity and PLI.

At discharge, a low‐fat highly digestible diet (Hill's Prescription Diet i/d Low Fat) was routinely recommended for all dogs. Alternatively, dog owners could prepare a low‐fat diet themselves (bland chicken or turkey breast, with rice, potatoes or couscous until first re‐check, followed by a balanced low‐fat diet compiled by a nutritionist if indicated). When lipase was within RI and CDAS < 3, a low‐fat diet was no longer recommended. At all re‐checks, the type of diets fed was recorded as a commercial low‐fat diet, home‐cooked low‐fat diet, hydrolyzed diet, or other diet.

Lipase activity was measured in house (LIPC, Roche on Cobas c501, Roche Diagnostics). The assay RI at the beginning of the study (24–108 U/L [6]) was revised in 2022 and changed to 17–156 U/L (Laureen Peters, Clinical Central Laboratory, Vetsuisse Faculty Berne, Switzerland), and throughout this study the 17–156 U/L RI is used when analyzing results. PLI (RI, 0–200 μg/L as reported by the laboratory, and 0–216 μg/L as published [4]) was measured at IDEXX, Switzerland. PLI values > 1500 μg/L are reported as 1500 μg/L by the laboratory.

Lipase activity and PLI from all re‐checks were measured from the same blood sample, whereas lipase activity and PLI at the time of diagnosis (*t*_1_) were not measured from the same blood sample in 17 dogs, all of which had markedly increased lipase results at admission. In these 17 dogs, PLI measurements (same laboratory) had already been initiated by referring veterinarians, and results were accepted as *t*_1_ values when the time interval between prereferral PLI measurement and lipase activity measurement at admission was maximally 36 h.

The prospective part of the study was approved by the Cantonal Veterinary office of Zurich and conducted in accordance with guidelines established by the Animal Welfare Act of Switzerland. Informed consent was given by all dog owners. Owner permissions for usage of leftover serum samples for additional PLI measurements from dogs not entering the prospective part of the study were also available.

### 2.2 Statistical Analyses

Data are reported as medians and ranges. Spearman's rank correlation coefficients (*r*_s_) were determined between lipase activity and PLI, at baseline (*t*_1_) and re‐checks. Spearman's rank correlation coefficients were also used to assess correlations among lipase activity, PLI, and CDAS. Fisher's exact test was used to assess associations between feeding low‐fat diets and lipase results categorized as “increased” and “within RI.” Mann–Whitney U tests were used when comparing age of dogs with follow‐up lipase results within RI versus above RI. Agreement between lipase activity and PLI (at > 200 and > 216 μg/L) above RI was assessed using Cohen's kappa coefficient.

Statistical analyses were performed using Graph Pad Prism software. For all analyses, a *p* < 0.05 was considered statistically significant.

## 3 Results

### 3.1 Dogs

A total of 106 dogs that had been hospitalized for suspected AP (*t*_1_) and seen again for a minimum of one follow‐up visit 2 weeks (*t*_2_) after discharge were included. Fifty‐six dogs were re‐checked after 6 weeks (*t*_3_), 24 dogs after 12 weeks (*t*_4_), and 13 dogs after 24 weeks (*t*_5_). During the study, data from seven dogs were excluded because of the occurrence of additional diseases (one dog each): aspiration pneumonia, esophagitis, pancreatic carcinoma, meningioma, copper‐associated hepatitis, steroid‐responsive meningitis, and polyarthritis, acute kidney injury.

The dogs' median age was 9 years (range, 1–17 years); median body weight was 11 kg (range, 1–44 kg), 50 (47%) dogs were female (39 spayed) and 56 (53%) dogs were male (39 neutered). The most frequently represented breeds were mixed‐breed (17), Yorkshire Terrier (six), French bulldogs (six), Labrador Retriever (five), Cocker Spaniel (four), Boxer (four), Dachshund (four), Malteser (three), Miniature Schnauzer (three), Prague Ratter (three), Chihuahua (three), Flat‐coated retriever (three), Golden Retriever (three), Pugs (three), and Poodle (two). Terrier breeds comprised 14/106 (13.2%) dogs. Abdominal ultrasound by a board‐certified radiologist was performed in 93/106 (88%) dogs, and 63/93 (68%) dogs had a US diagnosis of pancreatitis. The remaining 13 dogs without a full ultrasound had an abdominal focused assessment with sonography for trauma, triage, and tracking performed. Abdominal radiographs were taken in 73/106 (68.9%) dogs. All dogs without a full abdominal ultrasound by a radiologist had abdominal radiographs taken.

### 3.2 Results at the Time of Hospital Admission (*t*_1_)

Median lipase activity and PLI concentration in 106 dogs with suspected AP are shown in Table 3. Six dogs with lipase activities only slightly > 350 U/L (median 359 U/L, range 353–378 U/L) had concurrent PLI results ranging from 403 to 920 μg/L (median 439 μg/L). Three (2.8%) dogs with PLI < 400 μg/L (292, 343, 349 μg/L) had corresponding lipase activities of 424, 683, and 460 U/L. Lipase activities of 17 dogs with PLI measurements initiated by referring veterinarians ranged from 1320 to 11 530 U/L (median 2482 U/L). Sixteen of these 17 (94%) dogs had a PLI > 1500 μg/L; one dog had a PLI of 1248 μg/L.

Lipase activity and PLI at *t*_1_ correlated very strongly (*r*_s_ = 0.937, *p* < 0.0001; Table 1). Median CDAS at admission was 8 (range, 3–15). The most common clinical signs were lethargy (100/106, 94%), inappetence (97/106, 92%), vomiting (90/106, 85%), painful abdomen (76/106, 72%), and diarrhea (61/106, 58%). Median number of clinical signs was 3 (range, 2–6). Neither lipase activity nor PLI at *t*_1_ correlated significantly with CDAS or with the age of dogs.

**TABLE 1:** Spearman's rank correlation coefficient (*r*_s_) and statistical significance (*p* value) for correlations between lipase activity, PLI, and CDAS (clinical disease activity score) at the time of diagnosis (*t*_1_), and re‐checks *t*_2_ (2 weeks after discharge), *t*_3_ (6 weeks after discharge), *t*_4_ (12 weeks after discharge), and *t*_5_ (24 weeks after discharge).

Correlation between	*t*_1_ (*n* = 106)	*t*_2_ (*n* = 106)	*t*_3_ (*n* = 56)	*t*_4_ (*n* = 24)	*t*_5_ (*n* = 13)
Lipase activity and PLI	*r*_s_ = 0.937	*r*_s_ = 0.882	*r*_s_ = 0.918	*r*_s_ = 0.863	*r*_s_ = 0.911
	*p* < 0.0001*	*p* < 0.0001*	*p* < 0.0001*	*p* < 0.0001*	*p* < 0.0001*
Lipase activity and CDAS	*r*_s_ = 0.129	*r*_s_ = 0.391	*r*_s_ = 0.118	*r*_s_ = 0.113	*r*_s_ = 0.603
	*p* = 0.186	*p* = 0.0009*	*p* = 0.384	*p* = 0.6	*p* = 0.032*
PLI and CDAS	*r*_s_ = 0.111	*r*_s_ = 0.279	*r*_s_ = 0.142	*r*_s_ = 0.197	*r*_s_ = 0.570
	*p* = 0.257	*p* < =0.004*	*p* = 0.296	*p* = 0.357	*p* = 0.045*

*Note:* An alpha level of 0.05 was used to determine statistical significance. *denotes a signifcant *p* value.

### 3.3 Results at 2 Weeks After Discharge (*t*_2_)

Median lipase activity of 106 dogs 2 weeks (range, 13–16 day) after discharge was 91 U/L (range, 17–1920 U/L), median PLI was 95 μg/L (range, 30–1247 μg/L). Correlation between both lipase assays remained very strong (Table 1). Median CDAS was 1 (range, 0–7). A significant correlation between CDAS and both lipase activity (*r*_s_ 0.391, *p* = 0.0009) and PLI (*r*_s_ 0.279, *p* = 0.0038) was found. Lipase activity (*r*_s_ = 0.311, *p* = 0.001) and PLI (*r*_s_ = 0.326, *p* = 0.0007) correlated significantly with the dogs' age at *t*_2_. Dogs with still increased lipase activity and PLI were significantly (*p* = 0.0073) older (median 10 years versus median 9 years) compared to dogs with lipases within RI, while there was no difference in body weight.

Seventy‐seven of 106 (72.6%) dogs had both lipases within RI (Table 3). CDAS of 0 and 1 was recorded for 56/77 dogs (72.7%); CDAS distribution is given in Table 2.

**TABLE 2:** Overview of the clinical disease activity score (CDAS) in relation to lipase activity and PLI within RI or >RI at respective time points. For re‐checks, only dogs with both lipase assay results either within or >RI are depicted. CDAS of dogs with discordant lipase results in relation to respective RIs are described in the text. Re‐checks *t*_2_ (2 weeks after discharge), *t*_3_ (6 weeks after discharge), *t*_4_ (12 weeks after discharge), and *t*_5_ (24 weeks after discharge) RI lipase activity, 17–156 U/L; RI PLI, 0–200 U/L.

	*t*_1_ 106 dogs with concordant lipase assay results	*t*_2_ 101 dogs with concordant lipase assay results	*t*_3_ 55 dogs with concordant lipase assay results	*t*_4_ 22 dogs with concordant lipase assay results	*t*_5_ 13 dogs with concordant lipase assay results
CDAS of dogs (*n*) with lipase activity and PLI within RI	**NA**	***n* = 77** 0 and 1: 56/77 (72.7%) 2: 12/77 (15.6%) 3: 6/77 (7.8%) > 3: 3/77 (3.9%)	***n* = 34** 0 and 1: 30/34 (88.2%) 2: 2/34 (5.8%) 3: 1/34 (2.9%) > 3: 1/34 (2.9%)	***n* = 17** 0 and 1: 15/17 (88.2%) 2: 1/17 (5.9%) 3: 0/17 (0%) > 3: 1/17 (5.9%)	***n* = 8** 0 and 1: 8/8 (100%) 2: 0/8 (0%) 3: 0/8 (0%) > 3: 0/8 (0%)
CDAS of dogs (*n*) with lipase activity and PLI > RI	***n* = 106** 0 and 1: 0/106 (0%) 2: 0/106 (0%) 3: 4/106 (3.8%) > 3:102/106 (96.2%)	***n* = 24** 0 and 1: 12/24 (50%) 2: 4/24 (16.6%) 3: 3/24 (12.5%) > 3: 5/24 (20.8%)	***n* = 21** 0 and 1: 14/21 (66.6%) 2: 0/21 (0%) 3: 2/21 (9.5%) > 3: 4/21 (19%)	***n* = 5** 0 and 1: 3/5 (60%) 2: 1/5 (20%) 3: 0/5 (0%) > 3: 1/5 (20%)	***n* = 5** 0 and 1: 3/5 (60%) 2: 1/5 (20%) 3: 0/5 (0%) > 3: 1/5 (20%)

Abbreviation: NA, not applicable.

Compared to lipase assay RIs, 5/106 (4.7%) dogs had discordant (i.e., one lipase assay result within RI, one lipase assay result > RI) results (Table 3). One dog had lipase activity slightly > RI (180 U/L) and PLI within RI (133 μg/L); the dog's CDAS was 0. Four other dogs had lipase activities within RI (median 131 U/L, range, 127–143 U/L) and slightly elevated PLI (median 237 μg/L, range, 202–272). Their median CADS was 2 (range, 0–4).

**TABLE 3:** Number (*n*, %) dogs from re‐checks *t*_2_ (2 weeks after discharge), *t*_3_ (6 weeks after discharge), *t*_4_ (12 weeks after discharge), and *t*_5_ (24 weeks after discharge) with concordant and discordant (i.e., one lipase assay result within RI, one lipase assay result > RI) lipase results in relation to respective lipase assay RI (RI lipase activity, 17–156 U/L; RI PLI, 0–200 μg/L^a^, and published [4] RI PLI 0–216 μg/L^b^).

	*t*_1_	*t*_2_	*t*_3_	*t*_4_	*t*_5_
Number (*n*) of dogs re‐checked	106	106	56	24	13
Lipase activity and PLI within RI (median, range)	0/106 (0%)	77/106 (72.6%) 66 U/L (17–156) 63 μg/L (30–200)	34/56 (60.7%) 64 U/L (19–148) 66 μg/L (30–175)	17/24 (70.8%) 65 U/L (31–138) 58 μg/L (30–188)	8/13 (61.5%) 63 U/L (29–84) 49 μg/L (30–143)
Lipase activity and PLI > RI (median, range)	106/106 (100%) 997 U/L (353–11 530) 1141 μg/L (292–1500)	24/106 (22.6%) 495 U/L (174–1952) 592 μg/L (211–1247)	21/56 (37.5%) 405 U/L (168–1579) 434 μg/L (202–1500)	5/24 (20.8%) 380 U/L (160–507) 558 μg/L (211–662)	5/13 (38.5%) 610 U/L (535–799) 877 μg/L (218–1038)
Discordant results^a^ Discordant results^b^	0/106 (0%) 0/106 (0%)	5/106 (4.7%) 3/106 (2.8%)	1/56 (1.7%) 1/56 (1.7%)	2/24 (8.3%) 1/24 (4.2%)	0 (0%) 0 (0%)
Lipase activity within RI,					
PLI > RI^a^ (PLI > RI^b^)	0 (0%) 0 (0%)	4 (3.8%) 2 (1.9%)	1/56 (1.7%) 1/56 (1.7%)	1/24 (4.2%) 1/24 (4.2%)	0 (0%) 0 (0%)
Lipase activity > RI, PLI within RI^a,b^	0 (0%)	1 (0.9%)	0 (0%)	0 (0%)	0 (0%)
Cohens kappa (95% CI)^a^ Cohens kappa (95% CI)^b^	1 (1–1) 1 (1–1)	0.883 (0.783–0.983) 0.952 (0.881–1)	0.962 (0.887–1) 0.962 (0.887–1)	0.780 (0.495–1) 0.895 (0.658–1)	1 (1–1) 1 (1–1)

**FIGURE 1:**
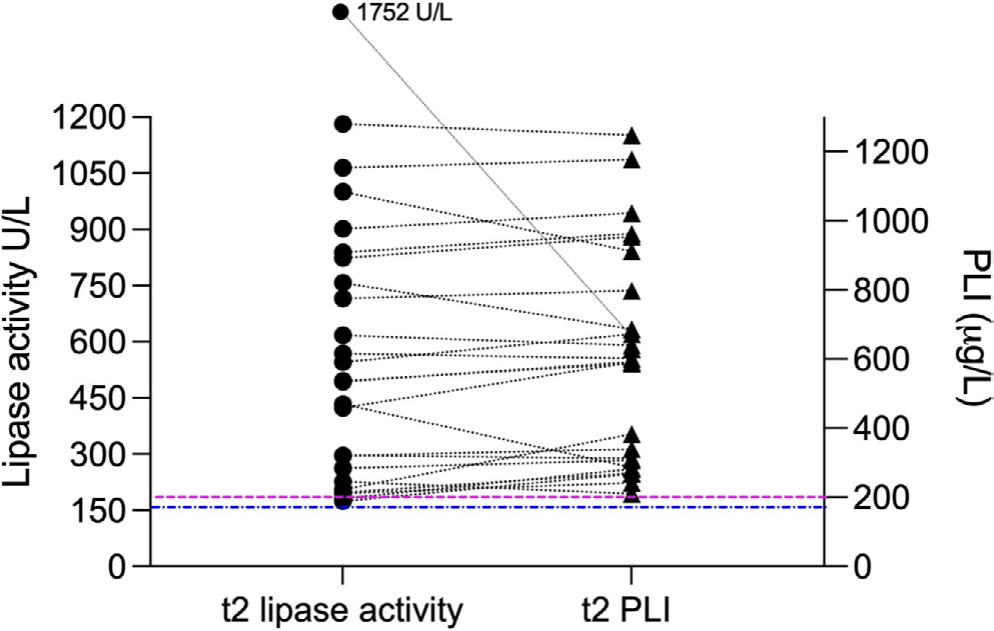
Abnormal lipase activity (dots) and PLI concentration (triangles) at 2 weeks after discharge from the hospital (*t*_2_; *n* = 24 dogs). For a clearer presentation, only dogs with lipase values > RI are shown here. Corresponding lipase values of dogs are connected with a dashed line. The blue line represents the lipase activity RI (17–156 U/L), the pink line represents the commonly used PLI RI (0–200 μg/L).

Twenty‐four (22.6%) dogs still had lipase activity and PLI > RI at *t*_2_ (Figure 1, Table 3). Twelve of 24 (50%) dogs had a score of 0 and 1; CDAS distribution is given in Table 2.

Types of diets fed at *t*_2_ are depicted in Table 4. Feeding a low‐fat diet (commercial low‐fat and home‐made combined) was not significantly associated with a lipase activity or PLI within RI. Lipase activity, PLI, and CDAS were also not significantly different between dogs fed a low‐fat diet (*n* = 84) and dogs not fed a low‐fat diet (*n* = 22).

**TABLE 4:** Overview of types of diets fed at different time points (*t*_2_ = 2 weeks after discharge), (*t*_3_ = 6 weeks after discharge), (*t*_4_ = 12 weeks after discharge), and (*t*_5_ = 24 weeks after discharge). Associations between a low fat diet (commercial and home‐made diets combined) and lipase activity (17–156 U/L) / PLI (0–200 mcg/L) within (w/i) or > RI were assessed using Fisher's exact test. An alpha level of 0.05 was used to determine statistical significance. The commercial low‐fat diet was Hill's Prescription Diet i/d Low Fat Digestive Care. Hydrolyzed diets comprised Royal Canin Anallergenic, Royal Canin Hypoallergenic, or Hill's Prescription Diet z/d.

	*t*_2_ *n* = 106 dogs	*t*_3_ *n* = 56 dogs	*t*_4_ *n* = 24 dogs	*t*_5_ *n* = 13 dogs
Types of diet fed (*n*, %)	Commercial low‐fat (66, 62.3%)	Commercial low‐fat (28, 50%)	Commercial low‐fat (10, 41.7%)	Commercial low‐fat (2, 15.4%)
	Homemade low‐fat (18, 17%)	Homemade low‐fat (9, 16.1%)	Homemade low‐fat (5, 20.8%)	Homemade low‐fat (4, 30.7%)
	Hydrolyzed (6, 5.7%)	Hydrolyzed (3, 5.3%)	Hydrolyzed (4, 16.7%)	Hydrolyzed (3, 23.1%)
	Other diet (16, 15%)	Other diet (16, 28.6%)	Other diet (5, 20.8%)	Other diet (4, 30.7%)
Association between low‐fat diet (y/n) and lipase activity w/i or > RI	*p* = 0.799	*p* = 0.567	*p* = 0.614	*p* = 0.266
Association between low‐fat diet (y/n) and PLI w/i or > RI	*p* > 0.999	*p* = 0.393	*p* = 0.999	*p* = 0.266

In three (3%) dogs, lipase activity and PLI had further increased at *t*_2_; their respective CDAS were 0, 1, and 7. Lipase activity and PLI remained elevated in two dogs with additional re‐checks beyond *t*_2_. Table S1 gives an overview of the increase (median, range) of both lipases at re‐checks.

### 3.4 Results at 6 Weeks After Discharge (*t*_3_)

Fifty dogs did not return for the 6‐week control. Of these, 40 (80%) had both lipases within RI at *t*_2_, two (4%) dogs had one lipase assay result within RI and one minimally elevated, and eight (16%) had both lipases > RI at *t*_2_. Median lipase activity of 56 dogs at *t*_6_ was 101 U/L (range, 19–1579 U/L), median PLI was 136 μg/L (range, 30–1500 μg/L). Correlation between both lipase measurements remained very strong (Table 1). Median CDAS was 0 (range, 0–7). No significant correlation between CDAS and both lipase assay results was found (Table 1). As seen at *t*_2_, age correlated significantly with lipase activity (*r*_s_ = 0.323, *p* = 0.015) and PLI (*r*_s_ = 0.375, *p* = 0.004) at *t*_3_. Dogs with elevated lipase activity and PLI at *t*_3_ were significantly (*p* = 0.0282) older (median 11.5 years versus median 9 years) compared to dogs with lipases within RI.

Thirty‐four dogs (60.7%) had both lipase assay results within RI (Table 3). A clinical score of 0 and 1 was recorded for 30/34 (88%) dogs; CDAS distribution is given in Table 2.

One dog had discordant lipase assay results in relation to the respective assay RIs. Lipase activity was 149 U/L, PLI was 269 μg/L, CDAS was 2.

**FIGURE 2:**
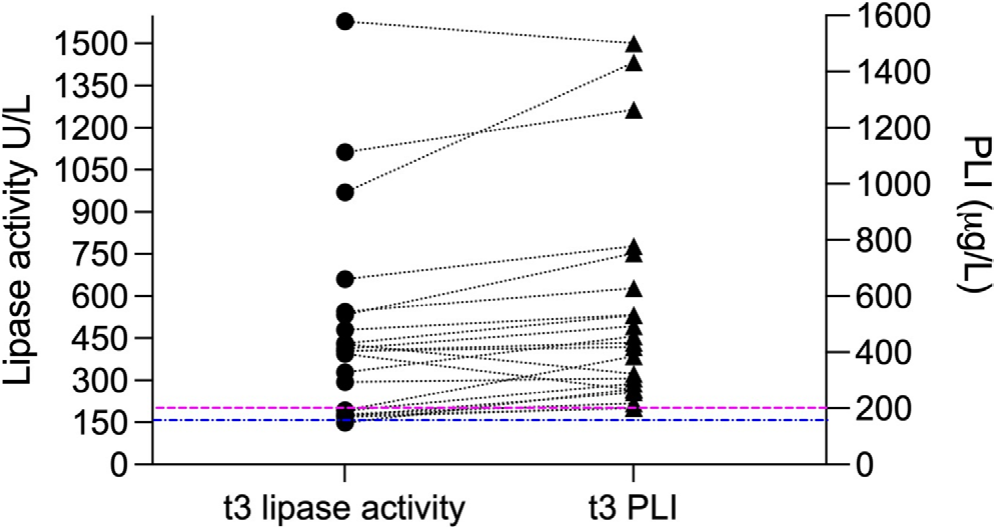
Abnormal lipase activity (dots) and PLI concentration (triangles) at 6 weeks after discharge from the hospital (*t*_3_; *n* = 21 dogs). For a clearer presentation, only dogs with lipase values > RI are shown here. Corresponding lipase values of dogs are connected with a dashed line. The blue line represents the lipase activity RI (17–156 U/L), the pink line represents the commonly used PLI RI (0–200 μg/L).

Twenty‐one (37.5%) dogs had increased results of both lipase assays (Figure 2, Table 3). Fourteen of 21 dogs with lipases > RI (66.6%) had a CDAS of 0 or 1; CDAS distribution is given in Table 2.

Types of diets fed at *t*_3_ are depicted in Table 4. Feeding a low‐fat diet (commercial low‐fat and home‐made combined) was not significantly associated with a lipase activity or PLI within RI. Lipase activity, PLI, and CDAS were also not significantly different between dogs fed a low‐fat diet (*n* = 37) and dogs not fed a low‐fat diet (*n* = 19).

In 10/56 dogs (18%), lipase activity and PLI had both increased again compared to 4 weeks earlier (*t*_2_). Their median CDAS was 0 (range, 0–6).

### 3.5 Results at 12 Weeks After Discharge (*t*_4_)

Thirty‐two dogs did not return to the 12‐week control. Of these, 20 (62.5%) had both lipases within RI at *t*_3_, one (3.1%) dog had one lipase assay result within RI and one minimally elevated, and 11 (34.4%) dogs had both lipases > RI at *t*_3_.

Median lipase activity of 24 dogs 12 weeks after discharge was 90 U/L (range, 31–507 U/L); median PLI was 119 μg/L (range, 30–662 μg/L). Correlation between both measurements remained very strong (Table 1). Median CDAS was 0 (range, 0–6). No significant correlations between CDAS and both lipase assay results were found. Age of dogs did not correlate significantly with lipase activity and PLI. Ages of dogs with elevated lipase activity and PLI were not significantly different compared to dogs with lipases within RI.

Seventeen of 24 (71%) dogs had both lipase activity and PLI within RI (Table 3). CDAS of 0 and 1 was recorded for 15/17 (88.2%) dogs; the distribution of CDAS is given in Table 2.

Two dogs (8.3%) had discordant lipase assay results in relation to the RI (Table 3). Lipase activity and PLI were 63 U/L and 255 μg/L (CDAS 0), and 113 U/L and 212 μg/L (CDAS 1) in these two dogs.

**FIGURE 3:**
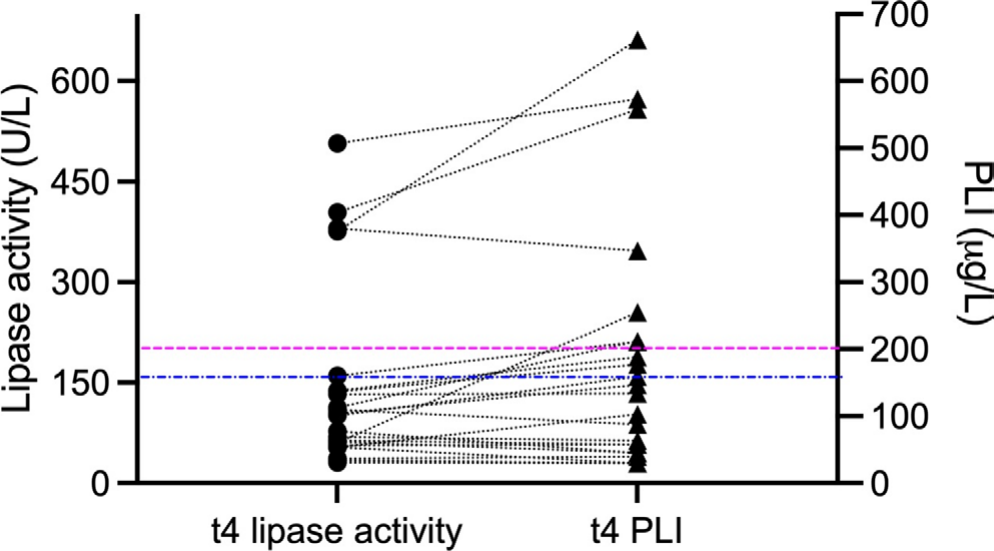
Lipase activity (dots) and PLI concentration (triangles) at 12 weeks after discharge from the hospital (*t*_4_; *n* = 24 dogs). Corresponding lipase values of dogs are connected with a dashed line. The blue line represents the lipase activity RI (17–156 U/L), the pink line represents the commonly used PLI RI (0–200 μg/L).

Five of 24 (20.8%) dogs had elevated lipase activity and PLI results at *t*_4_ (Figure 3, Table 3). Distribution of CDAS is given in Table 2.

Types of diets fed at *t*_4_ are depicted in Table 4. Feeding a low‐fat diet (commercial low‐fat and home‐made combined) was not significantly associated with a lipase activity or PLI within RI. Lipase activity, PLI, and CDAS were also not significantly different between dogs fed a low‐fat diet (*n* = 15) and dogs not fed a low‐fat diet (*n* = 9).

### 3.6 Results at 24 Weeks After Discharge (*t*_5_)

Eleven dogs did not return to the 24‐week control. All 11 (100%) had both lipases within RI at *t*_4_.

Median lipase activity of 13 dogs 24 weeks after discharge was 79 U/L (range, 41–799 U/L), median PLI was 133 μg/L (range, 30–1038 μg/L). Correlation between both lipase measurements remained very strong (Table 1). Median CDAS was 0 (range, 0–4). Both lipase results correlated significantly with CDAS (Table 1). Age did not correlate significantly with lipase activity and PLI at *t*_5_. Ages of dogs with elevated lipase activity and PLI at *t*_5_ were not significantly different compared to dogs with lipases within RI at *t*_5_.

**FIGURE 4:**
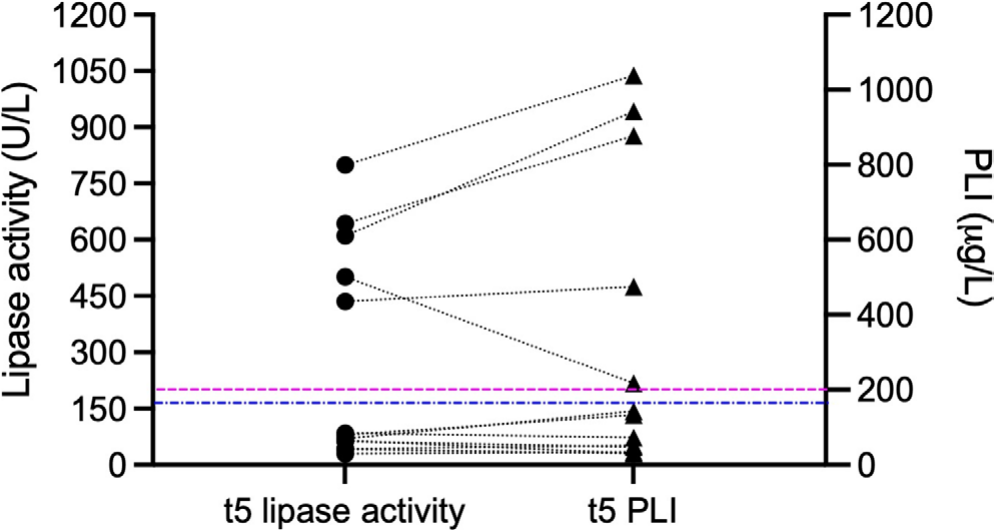
Lipase activity (dots) and PLI concentration (triangles) at 24 weeks after discharge from the hospital (*t*_5_; *n* = 13 dogs). Corresponding lipase values of dogs are connected with a dashed line. The blue line represents the lipase activity RI (17–156 U/L), pink line represents the commonly used PLI RI (0–200 μg/L).

Eight of 13 (61.5%) dogs had both lipase activity and PLI within RI at *t*_5_ (Table 3). A CDAS of 0 and 1 was recorded for all eight dogs. Five of 13 (38.5%) dogs had elevated lipase levels at *t*_5_ (Figure 4, Table 3). Three of five dogs (60%) with lipases > RI had a CDAS of 0 or 1; CDAS distribution is given in Table 2. All dogs with elevated lipase results at *t*_5_ also had elevated values at the previous three re‐checks.

Types of diets fed at *t*_5_ are depicted in Table 4. Feeding a low‐fat diet (commercial low‐fat and home‐made combined) was not significantly associated with a lipase activity or PLI within RI. Lipase activity, PLI, and CDAS were also not significantly different between dogs fed a low‐fat diet (*n* = 6) and dogs not fed a low‐fat diet (n7).

## 4 Discussion

In this study, the progression of clinical signs, lipase activity, and PLI in 106 dogs after hospitalization for suspected AP is described. All dogs were re‐checked 2 weeks after discharge; in addition, we were able to follow up on a subset of dogs up to 24 weeks after discharge. At the time the prospective part of the study was started, it was claimed that DGGR‐based lipase assays are less specific than the PLI test [15, 22]. This has since changed, and the company that markets PLI (Spec PL) now also offers a DGGR‐based lipase activity test that is marketed as a measurement of pancreatic lipase activity [23]. Since it is almost impossible to determine the specificity of a diagnostic test in the absence of a practicable gold standard, the idea at the time was to show that whenever lipase activity decreases or normalizes over time, PLI decreases or normalizes in the same way.

This is the largest follow‐up study to date after treatment for suspected AP in dogs and allows more insight into the further course of disease after hospitalization. The only study reporting follow‐up data after a suspected AP episode describes 16 dogs that were re‐checked at 2, 7, and 28 days after study enrollement [18]. While all dogs had normalized PLI results 28 days after enrollment, and no or only minimal clinical signs based on a similar clinical severity score, it was not reported how many dogs still had abnormal PLI results at earlier re‐checks, which makes a comparison with the data of this study difficult [18]. PLI results were not compared to the clinical status in that study [18]. In contrast, the focus of this study was how both lipase assay results compare to clinical signs over time when dogs are re‐examined.

At *t*_1_, a variety of clinical presentations and severity levels were included, reflecting the known wide spectrum of clinical severity levels seen in AP [18, 19, 21]. Not all dogs had ultrasonographic evidence of pancreatitis. It is known that pancreatic ultrasonography can be negative in the acute stages in dogs, as well as in people with AP [2, 3, 24, 25]. More importantly, no other disease was found that could have explained the presenting clinical signs and hyperlipasemia, and all dogs in this study had been hospitalized for a working diagnosis of AP. However, in the absence of a feasible diagnostic gold standard, we use the term “suspected AP” in this study.

During the course of the study, our lipase activity RI was revised. We had started the study using the RI 24–108 U/L [6, 7], which was replaced by a new RI (17–156 U/L) based on 123 healthy dogs (also measured on Cobas c501, LIPC Roche Diagnostics). This explains why a lipase cutoff of > 350 U/L (approximately three times > the upper limit of the previous RI) was the inclusion criterion of this study. In two previous regression analyses, approximately 350 U/L (355 U/L [10], and 348 U/L [6], same Roche LIPC assay) corresponded to the 400 μg/L PLI cutoff which is widely interpreted as “consistent with pancreatitis” [6, 10]. Since the new slightly higher RI, our threshold for an AP diagnosis is accordingly around 450 U/L. However the overwhelming majority of dogs in this study clearly had higher lipase activities than 350–450 U/L. Only 1/106 dog had a lipase activity slightly < 450 U/L (424 U/L) and a PLI < 400 μg/L (292 μg/L) at *t*_1_. This dog also had an ultrasonographic diagnosis of AP.

The PLI test specifically measures the concentration of pancreatic lipase, using a species‐specific and antipancreatic lipase specific antibody and has been found to be highly specific in studies using pancreatic histopathology as a diagnostic gold standard [14]. Lipase activity was measured using a catalytic test where the chromogenic substrate DGGR is believed to be selectively cleaved by pancreatic lipase [26]. The assay includes bile salts, colipase, and calcium chloride to optimize the enzymatic activity of pancreatic lipase [26]. Objection has been raised that DGGR‐based lipase assays are not specific for pancreatic lipase and subject to interference from nonpancreatic lipases in healthy dogs without pancreatitis [15]. The question remains what clinical relevance minimal deviations of lipase test results from each other have in a healthy state. Both assays used in this current study have been shown to decrease at the same rate during the first days of hospitalization for AP but were not compared to clinical signs in that study [6]. Our results now show that both assays also correlate highly when followed up over longer periods when the majority of dogs have no clinical signs anymore. Nature of change was always the same for both assays. Discordant results compared to RIs were rare. In those few cases, lipase activity was more often within RI while PLI results were still slightly elevated between 201 and a maximum of 272 μg/L. In this context it is important to note that the basis for this “questionable PLI range of 200‐400 μg/L” is unknown. This complicates comparisons of assay results, especially when lipase activity is within RI and PLI slightly > 200 μg/L. When comparing assays, dog populations behind respective lipase RI should also be considered. The current lipase activity RI is based on 123 healthy pet dogs of 54 different breeds with ages between 1 and 12 years, while the PLI RI is based on 93 healthy kennel dogs (“medium‐sized nonpurebred hounds”) of unknown ages [4]. The published upper PLI RI limit based on the upper 97.5th percentile of results of healthy dogs is in fact 216 μg/L [4]. Median PLI concentration was 39.4 μg/L with a range up to 275.0 μg/L [4]. Since individual breeds can have significantly higher PLI values (as shown for Dogues de Bordeaux) [27], it is conceivable that PLI values > 200 μg/L can also be found in healthy dogs. If compared to the published upper PLI limit of 216 μg/L [4] then only 3/106 dogs (2.8%) at *t*_2_, 1/56 (1.7%) at *t*_3_, and 1/24 dogs (4.2%) at *t*_4_ had discordant results (Table 3) in our study.

Clinical signs expressed as a disease activity score were also compared with lipase results and significant correlations were found at *t*_2_ und *t*_5_. Both lipase assays did not differ when compared to clinical severity. Lack of correlation between clinical severity and lipases at the time of diagnosis (*t*_1_) has not been described before. The fact that severely increased PLI values above 1500 μg/L are reported as 1500 μg/L might have contributed to the lack of correlation here, but results were the same for lipase activity. We think this is because the magnitude of hyperlipasemia can vary greatly in dogs with AP [6, 18, 19], also dogs can present clinically very differently as has been shown before [18, 19, 21]. Lack of correlation at *t*_3_ and *t*_4_ is likely due to the high number of clinically normal dogs with lipase values varying within low ranges but also clinically normal dogs with still elevated lipases. The number of clinically affected dogs with elevated lipases was quite low. At *t*_5_, there was again a positive significant correlation between lipase activity, PLI and CDAS. We assume this is because only dogs with clinically relevant disease (i.e., clinical signs and hyperlipasemia) as well as clinically normal dogs without lipase increases from dedicated owners were re‐checked after 6 months.

We decided to combine CDAS results of 0 and 1 because any (even minimal) weight loss results in 1 point in the original CIBDAI. Similar to other studies [18, 19, 20, 21] the basis of our CDAS was the CIBDAI. A CIBDAI up to 3 is considered clinically insignificant [17]. As our CDAS was additionally supplemented with information on abdominal pain, the 0–3 range seemed an even more cautiously formulated cut‐off. The large majority of dogs with lipases within RI had a low CDAS of 0–3. When applying a stricter standard (CDAS 0 and 1), still 72.7% of dogs with normal lipases had virtually no clinical signs 2 weeks after discharge, and this value increased over time until finally reaching 100% 24 weeks after discharge, albeit in a smaller sample. Conversely, the majority of dogs with elevated lipase activity and PLI at re‐checks had little or no clinical signs. This could be dogs that transitioned to chronic pancreatitis after an AP episode at *t*_1_ or dogs that have had recurrent pancreatitis episodes for some time. In order to make this distinction, we would need to know whether AP at *t*_1_ was actually the first episode, and we do not have that data. It is possible that some dogs have had pre‐existing chronic pancreatitis and were hospitalized for an acute on chronic pancreatitis episode, and subsequent lipase results cannot be directly related to the one AP episode in the hospital. The fact that lipase activity and PLI correlated significantly with age of dogs during re‐checks with a larger number of dogs (*t*_2_ and *t*_3_) is an indication that older dogs either take longer to normalize pancreatic lipase after a suspected AP episode or that older dogs are generally more likely to have chronic subclinical pancreatitis. We cannot compare our result to the literature, as it is still unknown how often serum lipase is elevated in the absence of clinical signs.

In all cases a low‐fat diet was recommended at discharge and 79% of dogs were fed a low‐fat diet at *t*_2_, 66% at *t*_3_, 63% at *t*_4_, and 46% at *t*_5_. Common reasons for not feeding a low‐fat diet were claims of intolerance to poultry, attachment to or dislike of a particular food brand by owners, or that dogs did not like eating it. Many owners of dogs with lipases within RI continued feeding a low‐fat diet, even if this was not recommended anymore. We could not detect a significant association between feeding a low‐fat diet and lipase activity or PLI within or above RI at *t*_2_ and later time points. However, this study was not primarily designed to study the effect of diet on pancreatic health. A study comparing equal‐sized groups where one low‐fat diet is compared to one control diet is better suited to investigate this. Since nothing has been published on this subject so far, we wanted to include this data here.

This study had some limitations. Ideally, all 106 dogs from *t*_1_ would have been followed up to *t*_5_. It turned out that this was not feasible because many clinically healthy dogs with lipases within RI or only minimally elevated did not return for further re‐checks. The Covid pandemic made check‐ups even more difficult. We assume that it was mainly dedicated owners who continued to come back for re‐checks, which is not necessarily related to the dogs' clinical condition.

Another limitation was the possibility of concurrent chronic enteropathies, which would be expected to be associated with similar clinical signs. However, it is difficult to exclude chronic enteropathies because clinical signs can be silent for long periods, clinicopathologic findings are nonspecific, ultrasonographic changes have low sensitivity for chronic enteropathy, and the absence of lymphoplasmacytic infiltrates on biopsies does not reliably rule out chronic enteropathies [28, 29, 30]. A small percentage of dogs were fed a hydrolyzed diet. Table S1 shows lipase and CDAS results of these dogs. These are likely dogs with diet‐responsive enteropathy; the majority had no or little clinical signs during re‐checks. It has recently been suggested that dogs with AP rarely have concurrent intestinal disease [21], but the prevalence of concurrent chronic enteropathy in dogs with AP is unknown.

In conclusion, no difference between both lipase tests when followed up over time, also not in relation to the clinical status of dogs was found. Almost a quarter of dogs still had elevated lipase activity and PLI 2 weeks after discharge, and this value reached over a third of dogs in the further course of the study. The large majority of dogs with elevated lipases at re‐checks had little or no clinical signs, and it remains to be seen whether or not lipase measurement in dogs without clinical signs provides any useful information. Future studies are needed to examine whether dogs with ongoing hyperlipasemia are the ones with a history of previous episodes of pancreatitis. Further study is also needed to examine the effect of a low‐fat diet on the clinical condition of dogs when recovering from AP.

## Disclosure

Author declares no off‐label use of antimicrobials.

## Ethics Statement

The study was approved by the Veterinary office of the canton of Zurich (national no 33602). Author declares human ethics approval was not needed.

## Conflicts of Interest

The author declares no conflicts of interest.

## Supporting information


**Data S1.** Supporting Information.

**Table S1.** Number (*n*, %) of dogs fed a hydrolyzed diet from re‐checks *t*_2_ (2 weeks after discharge), *t*_3_ (6 weeks after discharge), *t*_4_ (12 weeks after discharge), and *t*_5_ (24 weeks after discharge) with concurrent lipase activity (RI, 17–156 U/L) and PLI (RI, 0–200 μg/L) results, as well as their clinical disease activity score (CDAS).
