# Supplementary figures and images for: Evaluation of Hyperlipasemia and Clinical Signs in 106 Dogs After Hospitalization for Acute Pancreatitis: Results From a Combined Retrospective and Prospective Follow‐Up Study
Source: J Vet Intern Med. 2025 Aug 1;39(5):e70188. doi: 10.1111/jvim.70188 (PMC12314540; doi:10.1111/jvim.70188)

Scatterplot t2 lipase activity - CDAS

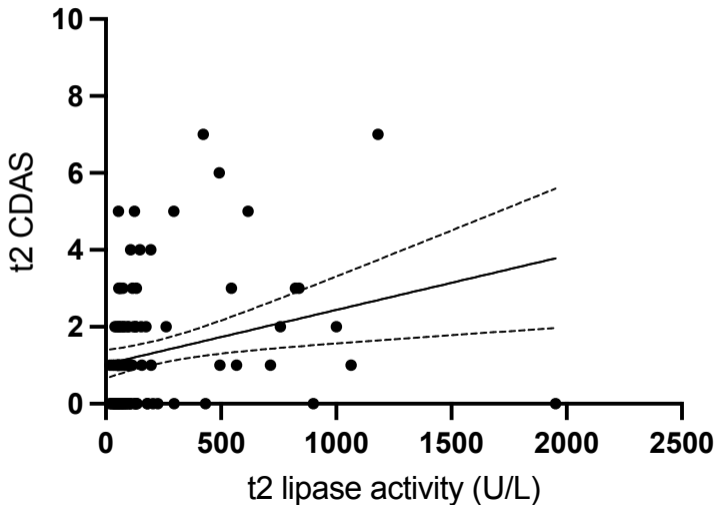

Supplement: Supplementary file 1 — Data S1. Supporting Information. [file JVIM-39-e70188-s002.pdf]
