# Supplementary material for: Evaluation of Hyperlipasemia and Clinical Signs in 106 Dogs After Hospitalization for Acute Pancreatitis: Results From a Combined Retrospective and Prospective Follow‐Up Study
Source: J Vet Intern Med. 2025 Aug 1;39(5):e70188. doi: 10.1111/jvim.70188 (PMC12314540; doi:10.1111/jvim.70188)
Supplement: Supplementary file 2 — Table S1. Number (n, %) of dogs fed a hydrolyzed diet from re‐checks t 2 (2 weeks after discharge), t 3 (6 weeks after discharge), t 4 (12 weeks after discharge), and t 5 (24 weeks after discharge) with concurrent lipase activity (RI, 17–156 U/L) and PLI (RI, 0–200 μg/L) results, as well as their clinical disease activity score (CDAS). [file JVIM-39-e70188-s001.docx]

Table S1: Number (n, %) of dogs fed a hydrolyzed diet from re-checks t2 (2 weeks after discharge), t3 (6 weeks after discharge), t4 (12 weeks after discharge), and t5 (24 weeks after discharge) with concurrent lipase activity (RI, 17-156 U/L) and PLI (RI, 0-200 µg/L) results, as well as their clinical disease activity score (CDAS).

|  | T2 | T3 | T4 | T5 | |
| --- | --- | --- | --- | --- | --- |
| Number (%) of dogs | 6/106  (5.7%) | 3/56  (5.3%) | 4/24  (16.7%) | 3/13  (23.1%) | |
| Lipase activity / PLI (CDAS) | 37 U/L / 30 µg/L (0)  70 U/L / 32 µg/L (0)  124 U/L / 116 µg/L (5)  153 U/L / 85 µg/L (2)  174 U/L / 268 µg/L (2)  1952 U/L / 629 µg/L (0) | 62 U/L / 75 µg/L (0) 132 U/L / 133 µg/L (4)  394 U/L / 266 µg/L (1) | 54 U/L / 30 µg/L (0)  138 U/L / 188 µg/L (0)  376 U/L / 662 µg/L (0)  507 U/L / 573 µg/L (0) | 62 U/L / 47 µg/L (0)  501 U/L / 217 µg/L (1)  643 U/L / 877 µg/L (1) | |
| Number (%) of dogs with lipases > RI | 2/6 (30%) | 1/3 (33.3%) | 2/4 (50%) | 2/3 (66.6%) |  |
